# A Multi-Model Approach to Implement a Dynamic Shelf Life Criterion in Meat Supply Chains

**DOI:** 10.3390/foods10112740

**Published:** 2021-11-09

**Authors:** Antonia Albrecht, Maureen Mittler, Martin Hebel, Claudia Waldhans, Ulrike Herbert, Judith Kreyenschmidt

**Affiliations:** 1 Institute of Animal Science, University of Bonn, Katzenburgweg 7-9, 53115 Bonn, Germany; maureen.mittler@uni-bonn.de (M.M.); martin.hebel@gmx.net (M.H.); c.waldhans@uni-bonn.de (C.W.); j.kreyenschmidt@uni-bonn.de (J.K.); 2 Research and Transfer Center, Anhalt University of Applied Sciences, Hubertus 1a, 06366 Köthen, Germany; Ulrike.Herbert@hs-anhalt.de; 3 Department of Fresh Produce Logistics, Hochschule Geisenheim, Von-Lade-Strasse 1, 65366 Geisenheim, Germany

**Keywords:** predictive microbiology, dynamic shelf-life, meat quality, meat spoilage, sensory modeling, food waste prevention

## Abstract

The high perishability of fresh meat results in short sales and consumption periods, which can lead to high amounts of food waste, especially when a fixed best-before date is stated. Thus, the aim of this study was the development of a real-time dynamic shelf-life criterion (DSLC) for fresh pork filets based on a multi-model approach combining predictive microbiology and sensory modeling. Therefore, 647 samples of ma-packed pork loin were investigated in isothermal and non-isothermal storage trials. For the identification of the most suitable spoilage predictors, typical meat quality parameters (pH-value, color, texture, and sensory characteristics) as well as microbial contamination (total viable count, *Pseudomonas* spp., lactic acid bacteria, *Brochothrix thermosphacta*, Enterobacteriaceae) were analyzed at specific investigation points. Dynamic modeling was conducted using a combination of the modified Gompertz model (microbial data) or a linear approach (sensory data) and the Arrhenius model. Based on these models, a four-point scale grading system for the DSLC was developed to predict the product status and shelf-life as a function of temperature data in the supply chain. The applicability of the DSLC was validated in a pilot study under real chain conditions and showed an accurate real-time prediction of the product status.

## 1. Introduction

Dynamic shelf-life modeling is an emerging field within the sector of predictive microbiology of foods. With detailed knowledge of the spoilage kinetics of a specific product, a real-time prediction of the product status is possible based on data of the environmental conditions in the supply chain [1]. Thus, the combination of sensor data and predictive models is one innovative strategy to support cold chain management and reduce food waste [2]. This is mainly performed by optimizing logistic decisions, display rotation at the retailer site, dynamic pricing, and an efficient use of the sales window [3,4,5]. The need to reduce food waste is urgent for perishable products with a high use of resources, such as meat. Food waste occurring in the fresh meat sector sums up to 23% of production [6]. A main cause is the high perishability, which leads to a short shelf-life and selling time [7] and improper handling of the products in the supply chain or at the consumer level [6,8]. Besides, meat products are marked by a high global warming potential (GWP) and cause the highest environmental impact among food products [8,9,10]. Thus, reducing food waste in the meat sector is an important task for meeting the 17 Sustainable Development Goals (SDGs), defined by the United Nations. The approach of dynamic modeling for specific products and supply chains can be used to realize this task.

Even though several attempts have been made to work out the conceptual frameworks of dynamic shelf-life modeling, some aspects hinder the practical implementation of these approaches in supply chains. Skepticism from the actors in the chain, legislative concerns and a lacking digitalization are main reasons delaying the practical application. In order to support the implementation, a mindset shift is required to realize the opportunities of monitoring and data exchange as a tool to establish best practices instead of a risk for goods return [11]. Thus, an integration of sensor data, smooth passing of information between the actors of the supply chain as well as proper data handling remains difficult though innovative solutions such as traceability technologies, interfaces and data management clouds are available [2,12].

The base for the combination of sensor data and predictive microbiology is built: over 700 models and extensive databases are available for food environments, however, only a few are implemented in software systems or applied in food supply chains to predict shelf-life [2]. Most of these tools have a strong focus on pathogen modeling by using growth or inactivation models as well as microbial risk assessment tools for evaluating the safety of food [13]. Experimental data underlying these predictions is often gained under laboratory conditions in broth or controlled food-microorganism combinations [14]. Since the conditions in real meat supply chains are characterized by complex food structures, microbial interactions, different packaging and environmental conditions, the applicability of these databases and models is sometimes limited [7,15]. For dynamic shelf-life predictions in real-time, an accurate modeling of the microbial growth of spoilage organisms as function of environmental parameters such as temperature is required [2,16]. This dynamic growth modeling approach provides successful predictions of the remaining shelf-life, shown for example, for aerobe packed products such as fresh pork and poultry filets under real chain conditions [5,14,17]. For achieving this goal, the identification and modeling of a specific spoilage organism (SSO) is crucial for the accuracy of the predictions because these organisms dominate the microbial growth and thus determine the deterioration and spoilage kinetics of the product [7]. Nevertheless, for the most commonly used packaging for fresh meat, such as modified atmosphere packaging (map) or vacuum packaging, the identification of the SSO remains difficult. The dominating microorganism can vary with temperature and thus hinders the modeling of remaining shelf-life based on microbial indicators [18,19]. Consequently, a match between microbial and sensory parameters is hindered when focusing on the SSO concept under these packaging conditions. Therefore, a combination of different spoilage indicators and the integration into an index is a useful approach in order to set limiting quality attributes for real chain conditions [1]. A revised and more accurate shelf-life prediction is then possible by a multi-quality approach based on the product specific shelf-life markers and the integration in mathematical tools [20,21,22].

Thus, the aim of the study was the development of a dynamic shelf-life criterion for fresh ma-packed pork and its integration in the meat supply chain via sensor data. The shelf-life criterion was assessed in a multi-model approach during storage trials with six constant and three dynamic temperature conditions. The validation of the models was conducted in two pilot studies under real chain conditions.

## 2. Materials and Methods

### 2.1. Samples and Experimental Design

The investigations focused on sliced fresh pork loins (*M. longissimus dorsi*) which were provided by a slaughtering and processing company in Germany. The samples were randomly selected from the production intended for commercial use and covered different fatteners and producers, respectively. Each sample package (400 g) contained five slices of pork loin (0.9–1.2 cm thick) packed under modified atmosphere (70% O_2_, 30% CO_2_). The samples were transported from the processing site to the laboratory (University of Bonn) under temperature-controlled conditions. The samples were packed in polystyrene boxes equipped with cooling pads to ensure an appropriate cooling during the transport (2.5 h) to the laboratory. In the laboratory, the samples were stored in low-temperature, high precision incubators (MIR 153, SANYO Electric Co., Ora-Gun, Gumma, Japan). The transportation and storage temperature was monitored by data loggers (Escort Junior Internal Temperature Data Logger, Escort, New Zealand), with measurements taken every ten minutes. During storage, the samples were analyzed for physicochemical, microbial, and sensory parameters at seven to twelve subsequent investigation points until the end of shelf-life was reached. First investigations started directly after arrival in the laboratory. The first investigation point comprised ten samples, while the following investigations were conducted with a sample size of five per investigation point and storage scenario.

For the development of a dynamic shelf-life model, a total of 14 storage trials were conducted which covered different storage scenarios. Samples for each trial were taken separately. In a first step (Trial A), six different isothermal storage conditions (2, 4, 7, 10, 15 and 25 °C) were investigated to describe the spoilage kinetics of the product and identify the most relevant quality parameters for the prediction of the spoilage process (Table 1).

**Figure 1:**
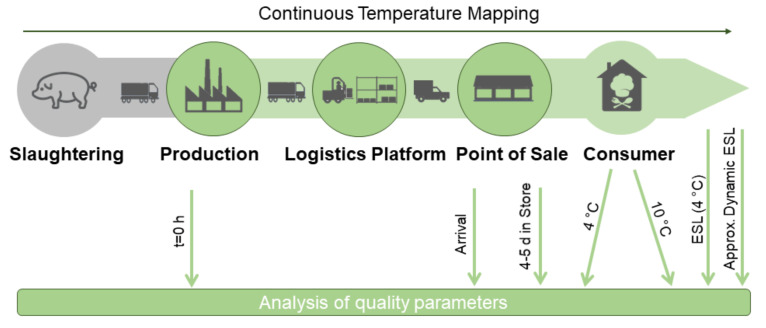
Scheme of the pilot study in a German pork supply chain (ESL—estimated shelf-life).

In a second step, three different storage scenarios (Trials B, C and D) at non-isothermal temperature conditions were conducted under laboratory conditions to validate the prediction of shelf-life. The non-isothermal storage scenarios were designed based on temperature data of former temperature monitoring studies in order to reflect real chain conditions. The dynamic trials covered temperature violations in the beginning (Trial B), during exponential growth (Trial C) and a real chain scenario with an end storage at 10 °C for simulating consumer refrigerators (Trial D). Every non-isothermal storage test was accompanied by a constant 4 °C reference. In a third step, the implementation into practice was validated with a pilot study in a German pork supply chain. Therefore, pork filets were tracked along their entire supply chain. The supply chain started at the slaughtering and production company, where the produced samples were transported via a logistics platform to a local supermarket. After the point of sale, the samples were stored at 4 °C and 10 °C, respectively in order to simulate consumer scenarios with typical fridge temperatures. Samples were taken at each point of the chain until the end of storage in order to investigate quality parameters and assess microbial contamination (Figure 1). In parallel, continuous temperature monitoring was carried out over the entire test period.

### 2.2. Physicochemical Quality Parameters

The pH-value was measured on the meat surface using a portable pH-meter (Five Go FG2/EL2, Mettler Toledo, Schwerzenbach, Switzerland). Three measurements were performed at different positions for each sample and the mean pH-value was calculated.

The CO_2_ and O_2_ concentration in the packaging were measured using a handheld gas analyzer (OXYBABY, WITT-Gastechnik GmbH & Co KG, Witten, Germany). Gas concentrations are given as volume percentages of the total packaging atmosphere. Gas analysis for each sample was carried out at every investigation point on the sealed packaging before opening the sample for microbiological and sensory analysis. Every sample was measured three times and the mean value was calculated.

The color was measured using a large view spectrophotometer (ColorFlex EZ 4500L, HunterLab, Murnau, Germany) with D65 illuminant (6500 K daylight) and a 45°/0° geometry, at a wavelength between 400 nm and 700 nm. The CIE 1976 L*a*b* scale was used (international standard, internal company software). The filets were placed on the glass surface of the device and measured at three sample points to obtain a representative evaluation of the sample. Based on the three sample points, a mean value was calculated for each sample.

For investigating the texture of the samples, a texture profile analysis (TPA) was conducted using a texture analyzer TA-XTplusC (Stable Micro Systems, Godalming, UK) with a 11.11 mm Magnus Taylor cylindric domed probe. The TPA is a standard procedure of the devices company software and comprises the parameters elasticity, cohesion, chewiness, hardness, gumminess, adhesive strength and springiness. For the measurement, 30% of the surface was compressed with a trigger force of 10.2 g and a test speed of 1 mm/sec. The measurements were conducted with three repetitions at different positions of the sample and the mean value was calculated for each sample.

### 2.3. Microbiological Parameters

For microbial analysis, a representative product sample (meat surface tissue with a size of 4.0 × 8.0 cm and a weight of 10 g) was extracted from the top slice in the package. The sample was transferred to a filtered stomacher-bag, filled with saline peptone diluents (0.85% NaCl with 0.1% peptone, Oxoid Ltd., Basingstoke, UK) to a final weight of 100 g and mixed for 60 s using a Stomacher 400 (Kleinfeld Labortechnik, Gehrden, Germany). Tenfold dilution series of the homogenate were prepared using saline peptone diluents. Appropriate dilutions were transferred to the different growth media in petri dishes.

Total viable count (TVC) was determined by pour-plate technique on plate count agar (Merck, Darmstadt, Germany). For assessing the mesophilic TVC and total psychrotroph count (TPC), the plates were incubated at 30 °C for 72 h (ISO 4833, product specifications) and at 20 °C for 120 h (internal laboratory SOP), respectively. *Pseudomonas* spp. (PSE) were detected by spread-plate technique on Pseudomonas agar with cetrimide fucidin cephalosporin (CFC) selective supplement (Oxoid Ltd., Hampshire, UK). The incubation period was 48 h at 25 °C. *Brochothrix thermosphacta* were identified by drop-plate technique on streptomycin inosit toluylene red agar supplemented with selective supplement (Oxoid Ltd., Hampshire, UK). Plates were incubated at 25 °C for 48 h. Enterobacteriaceae were identified by overlay treatment on violet red bile dextrose agar (VRBD) (Merck, Darmstadt, Germany) and incubation at 30 °C for 48 h. Lactic acid bacteria (LAB) were detected by pour-plate technique on de Man, Rogosa, Sharpe agar (MRS) (VWR Chemicals, Leuven, Belgium). Plates were incubated at 37 °C for 72 h according to the product specifications of the culture medium. Yeasts and molds were determined by spread-plate technique on Yeast extract glucose chloramphenicol agar (YGC) (Merck KGgA, Darmstadt, Germany) and incubation for 120 h at 25 °C. Each sample was analyzed and enumerated in duplicate. Resulting data were calculated as log_10_ CFU/g.

### 2.4. Sensory Analysis

A trained sensory panel (3–6 persons) from the Institute of Animal Science (University of Bonn) carried out the sensory investigations with a focus on the organoleptic assessment of the samples. The panelists evaluated the pork loins in the sealed package. Color, texture, and odor were evaluated using a 5-point scale with five meaning highest quality and freshness and one meaning unacceptable and spoiled. A sensory index (SI) was calculated as a weighted average with the following equation (Equation (1)).

(1) $$\mathrm{SI}=\frac{2\;\cdot \;O+2\;\cdot \;C+T}{5}$$

where SI is the corresponding evaluation at time *t* [h], *O* represents the evaluation of odor, *C* is the evaluation of the color and *T* is the texture.

In order to calculate the sensory shelf-life of the samples, the sensory acceptance level was set to 2.8 according to the assessment scheme.

### 2.5. Data Analysis and Modeling

A Spearman’s rank correlation was conducted using SPSS 24 (IBM Corporation 1989, 2013, New York, NY, USA) for identifying the parameters which are most relevant to predict the quality and shelf-life of the product. For every isothermal temperature in Trial A, the correlation of the parameters to storage time was investigated. The parameters for further modeling were selected based on very high correlations (r > 0.9) as indicated by the correlation coefficient r.

Microbial and sensory data were fitted based on the Levenberg–Marquardt algorithm using the statistical software program OriginPro 8.0G (OriginLab Corp., Northampton, MA, USA).

#### 2.5.1. Modeling of Microbial and Sensory Data

Primary (dependency from time) and secondary models (dependency from temperature) were combined following the approach of Kreyenschmidt et al. 2010 and Bruckner et al. 2012.

As primary model, to describe the microbial growth as a function of time, the modified Gompertz equation was used (Equation (2)) [23].

(2) $$N_{t}=A+C\;\cdot \;e^{-e^{-B\;\cdot \;\left(t-M\right)}}$$

where, *N_t_* is the count of microorganism [log_10_ CFU/g] at time *t* [h], *A* is lower asymptotic line of the growth curve (the initial count of microorganism [log_10_ CFU/g]), *C* is the difference between upper asymptotic line of growth curve and the lower asymptotic line of the growth curve [log_10_ CFU/g], *B* is the relative maximum growth rate at time *M* [1/h], *M* is time at which maximum growth rate is obtained (reversal point) [h] and *t* is time [h].

As primary model for the sensory data, the SI was plotted against time and fitted with a linear model for each isothermal storage trial (A).

(3) SI=m · t+b

where SI is the corresponding evaluation at time *t* [h], *m* represents the specific sensory spoilage rate and *b* is the maximum evaluation at storage time 0.

The influence of temperature on the relative growth rate *B* at time *M* (microbial data) was assessed using the Arrhenius equation as secondary model. *B* and *M* values obtained from the Gompertz function at different isothermal temperature scenarios were fitted linearly using Equations (4) and (5). For this approach, Trial A6 (25 °C) was excluded to avoid inaccuracies in the prediction of the lowest storage temperatures.

(4) $$\ln\left(B\right)=ln\left(F\right)-\left(\frac{E_{a}}{R\;\cdot \;T}\right)$$

where *B* is the relative growth rate at *M* [1/h], *F* is pre-exponential factor [1/h], *E_a_* activation energy for bacterial growth [kJ/mol], *R* is gas constant [8.314 J/mol/K], *T* is absolute temperature [K].

(5) $$\ln\left(M\right)=ln\left(F\right)-\left(\frac{E_{a}}{R\;\cdot \;T}\right)$$

where *M* is time at which maximum growth rate is obtained (reversal point) [h], *F* is pre-exponential factor [1/h], *E_a_* activation energy for bacterial growth [kJ/mol], *R* is gas constant [8.314 J/mol/K], *T* is absolute temperature [K].

To describe the influence of storage temperature on the sensory characteristics of the product, the specific sensory spoilage rates (*m*) at isothermal temperatures were plotted against the temperature *T* [K] and fitted with a linear Equation (6) as a secondary model.

(6) m=a · T+b

where *m* is the corresponding sensory spoilage rate at temperature *T* [K], *a* represents the slope and *b* is the y-axis intercept.

#### 2.5.2. Dynamic Modeling

For the dynamic model, primary and secondary models were combined as described by [14,24]. Shortly, the combined model predicts the microbial growth within separate isothermal intervals and combines the intervals to a coherent model. This approach is conducted via temperature-dependent modeling of the Microbial Growth Rate *B* and a recalculation of the inflection point *M* based on the microbial counts at the end of the previous temperature interval. The microbial growth is then predicted by using a composite Gompertz function including the growth parameters (*B* and *M*) delivered by secondary level modeling. For the microbial model, the following assumptions were defined: The first assumption was that the maximum of the microbiology count does not depend on temperature [15]. The second assumption was that the microbial count of the same product will achieve an equivalent plateau [24]. Therefore, the maximum bacterial count (*Nmax*) was set to the average of maximum concentrations obtained during the storage experiments at constant temperature scenarios.

For the sensory model, the combination of primary and secondary models is conducted equivalently. The slope of the sensory decay of each temperature interval was calculated via secondary modeling. The corresponding sensory evaluations for the first temperature interval can be calculated using both parameters (*b* and *m*). For all further intervals, *b* must be adjusted to ensure a seamless transition of the intervals. For this purpose, the SI of the products at the end of the previous interval needs to be calculated (7) and inserted to Equation (8).

(7) $$\mathrm{SI}\left(t\right)=m_{p}\;\cdot \;t_{p}+b_{0}$$

where SI(t) is the sensory index at the beginning of the interval, *m_p_* is the specific sensory spoilage rate of the previous interval, *t_p_* is the storage time of the products up to this interval and *b*_0_ is the maximum evaluation at storage time 0 of the previous interval.

(8) $$b_{adj}=\mathrm{SI}\left(t\right)-m\;\cdot \;t_{p}$$

where *b_adj_* is the adjusted maximum evaluation at storage time 0, SI(t) is the sensory index at the beginning of the interval, *m* is the specific sensory spoilage rate of the interval and *t_p_* is the storage time of the products up to this interval.

#### 2.5.3. Model Validation

The validation of the model prediction was conducted by comparing predicted vs. observed values of microbial and sensory data, respectively. The performance indices root mean square error (RMSE), coefficient of determination (R^2^), accuracy factor (Af) and bias factor (Bf) were calculated following the methodology od former studies [24,25].

As for the accuracy factor, a bias factor of 1.00 shows a perfect agreement between observed and predicted values. An underestimation (fail-dangerous) of microbial counts would lead to a bias factor above 1.00, an overestimation (fail-safe) to a bias factor below 1.00 [25].

## 3. Results and Discussion

### 3.1. Characterization of the Spoilage of Ma-Packed Pork Loin and Selection of the Shelf-Life Criterion

The analysis of quality and microbial parameters of the constant storage trials revealed that pork samples showed a high quality upon arrival at the laboratory. The mean pH of the filets was 5.64 ± 0.04, (*n* = 262) which is in accordance with former studies focusing on fresh pork filet [26]. The color of the filets was fresh red, and measurements showed a mean L*-value of 53.61 ± 1.21, an a*-value of 7.78 ± 1.02 and a b*-value of 16.27 ± 0.76. The measurements of the atmosphere in the packaging showed a mean O_2_-content of 74.48% ± 0.96 and CO_2_-content of 19.57% ± 0.61 directly after production. The texture profile analysis (TPA) revealed a mean hardness of 1.07% ± 0.18, an elasticity of 84.90% ± 6.26 and a cohesion of 59.09% ± 2.66 at the beginning of storage. The sensory analysis pointed to a high quality with a mean SI of 4.83 ± 0.07 upon arrival at the laboratory. The initial microbial contamination was 2.54 ± 0.52 log_10_ CFU/g for mesophilic and 2.74 ± 0.58 log_10_ CFU/g for TPC. The mean contaminations with PSE, LAB, Enterobacteriaceae, *B. thermosphacta* and yeasts and molds were under the detection limit. All quality parameters showed a pronounced deterioration during storage, which accelerated at higher storage temperatures. The dependency of different quality parameters on storage time is displayed in Table 2 for every isothermal storage trial. At the end of storage, the mean pH decreased to a value of 5.51 ± 0.11. An increase of pH-values was linked to the microbial spoilage process and the accumulation of metabolites, such as ammonium, in former studies [26,27]. However, the development of pH-values is related to the composition of microflora, interaction, and competition between microorganisms as well as the gas atmosphere of the packaging [28,29]. Thus, the decrease of pH-values in this trial can be explained both by the growth of LAB leading to an accumulation of lactic acid on the product and by the increase of carbon dioxide in the packaging. Since these processes relate to the temperature and velocity of microbial growth, but show no consistent pattern in the isothermal trials, the pH-value is unsuitable for dynamic shelf-life modeling of ma-packed pork filets in this trial.

During storage, the color of the filets changed to a paler and slightly greenish hue. The L*-value increased to a mean level of 59.77 ± 2.53, while the a*- and b*-value decreased to values of 5.02 ± 1.07 and 15.05 ± 0.97 respectively. The color measurements were characterized by high standard deviations at the respective investigation points. Since the most relevant change in the red color occurred at the beginning of storage, no acceptance level could be identified for an advanced spoilage process and the end of shelf-life. Additionally, the color measurements showed a high variation between batches. Thus, the color measurements are interpreted as relative rather than absolute values and are not suitable for overall shelf-life modeling. The measurements of the atmosphere inside the packaging showed a switch in the gas composition, with a mean O_2_ concentration of 10.24% ± 10.46 and CO_2_ of 82.37% ± 14.44, caused by the metabolic activity of growing microorganisms. These effects were also observed in other trials investigating ma-packed fresh meat, especially at the beginning of storage [30,31]. During the isothermal trials, a significant decrease of the O_2_ was observed, after TPC reached the acceptance limit of 7 log_10_ CFU/g. While some samples showed no changes in the atmosphere at all, measurement of other samples showed that O_2_ was completely consumed by microorganisms. Thus, measurements of the atmosphere showed a high variation covering the complete measuring range, which made the parameter unsuitable for shelf-life modeling. The results of the TPA showed a higher hardness, with a mean of 1.48% ± 0.38, and a reduced elasticity and cohesion, with values of 71.62% ± 3.22 and 56.46% ± 0.89, respectively. The sensory analyses resulted in a mean SI of 1.15 ± 0.16 at the last investigation point which is near to the lowest quality classification of one. The highest microbial count at the end of storage was observed for the TPC with a mean value of 8.29 ± 0.28 log_10_ CFU/g, while the mesophilic TVC reached a significantly lower mean level of 6.80 ± 1.26 log_10_ CFU/g. All bacteria examined showed growth over the storage period with maximum counts for LAB (5.37 ± 1.21 log_10_ CFU/g) and PSE (5.36 ± 2.06 log_10_ CFU/g) at the end of storage. Yeasts and molds increased to a mean maximum value of 5.03 ± 1.42 log_10_ CFU/g. Enterobacteriaceae remained below the detection limit for 2–4 °C and *B. thermosphacta* for 2, 4 and 10 °C. At 7, 10, 15 and 25 °C, microbial growth was observed for these germs, with Enterobacteriaceae showing mean germ counts of 5.00 ± 1.32 log_10_ CFU/g and *B. thermosphacta* reaching 5.79 ± 1.31 log_10_ CFU/g.

**Figure 2:**
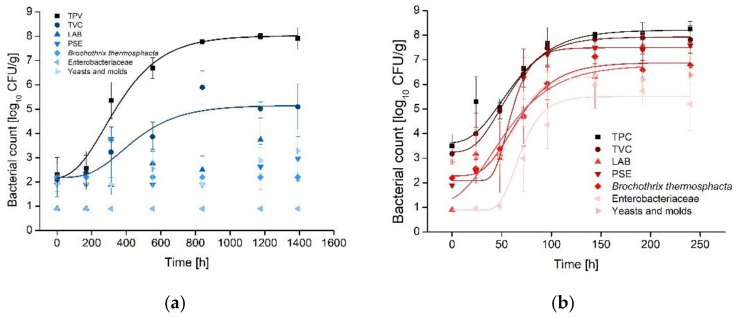
Growth of the total microbial populations and typical spoilage organisms on ma-packed pork loin stored at (**a**) 2 °C (mean ± standard deviation, *n* = 40), (**b**) 15 °C (mean ± standard deviation, *n* = 45), all growth curves fitted with modified Gompertz equation.

Differences between TVC and TPC at the stationary phase was low at higher temperatures (Δ 0.43 log_10_ CFU/g, 15 °C and Δ 0.09 log_10_ CFU/g, 25 °C), Figure 2. In contrast, the difference between both cultivation methods at the stationary phase was high at low storage temperatures (Δ 2.83 log_10_ CFU/g, 2 °C). Thus, the incubation temperature specified by classical enumeration techniques has a high impact on the results of the plate count, especially for cold storage conditions. Estimating the mesophilic TVC as stated in the legal regulations for fresh produce of animal origin, leads to a strong bias for perishable products stored at low temperatures. At the end of shelf-life, a mean underestimation of the contamination levels by 0.78 log_10_ CFU/g was reported before for fresh raw meat when comparing mesophilic (30 °C incubation) and psychrotroph (22 °C incubation) counts [32]. Thus, building models on the base of mesophilic TVC would lead to an underestimation of microbial growth at lower temperatures due to thermal selection processes during incubation of the plates at 30 °C.

Additionally, not all investigated germs displayed a typical sigmoidal growth curve and there was no reproducible pattern of organisms that grew dominant at all investigated temperatures (Figure 2). This effect was also described in former studies [18]. For future investigations, we propose cultivation methods adjusted for psychrotroph LAB as it was shown by [32] since this might show a more realistic representation of the spoilage processes occurring in fresh ma-packed meat stored at cooling conditions. Even though all investigated microorganisms showed a clear temperature dependent growth, the spoilage kinetics and microbial composition varied in dependency from the storage temperature. Thus, no SSO could be identified for a reliable prediction of microbial growth under different temperature conditions. Typically, SSO are selected to establish shelf-life models for fresh meat or meat products to ensure the most accurate predictions [7]. However, when no SSO can be identified, the combination of TVC with quality parameters is a promising approach for real-time prediction of dynamic shelf-life [33].

Since enumeration techniques focusing on TPC showed an accurate estimation of microbial growth and reached the acceptance limit of 7 log_10_ CFU/g at all isothermal experiments, TPC were selected for microbial modeling.

This finding was supported by the correlation analysis between quality parameters and storage time for all investigated temperatures. The highest correlations could be identified for the TPC (r ≥ 0.89) and for the sensory index (r ≥ 0.97) for all storage temperatures (Table 2 and Table 3).

**Table 2:** Correlation of the different quality parameters and the storage time of ma-packed pork loins under different isothermal conditions (Spearman’s rho).

	Texture			Color					
Temperature [°C]	Hardness	Elasticity	Cohesion	L*	a*	b*	pH	Oxygen	SI
2	0.341 *	−0.318 *		0.474 **	−0.712 **		−0.705 **		−0.951 **
4		−0.253 *		0.408 **	−0.671 **	−0.602 **	−0.526 **	−0.903 **	−0.949 **
7	0.549 **	−0.693 **	−0.622 **	0.663 **	−0.645 **	−0.724 **	−0.657 **	−0.881 **	−0.971 **
10				0.551 **	−0.692 **	−0.554 **	−0.558 **		−0.986 **
15	0.302 *	−0.605 **	−0.648 **	0.726 **	−0.571 **	−0.404 **		−0.948 **	−0.977 **
25		−0.700 **	−0.403 **	0.763 **			0.296 *	−0.964 **	−0.988 **

The correlation coefficient r is indicated only for significant correlations with * meaning significant at the 0.05-level and ** meaning significant at the 0.01-level.

According to Table 3, PSE, *B. thermosphacta*, Enterobacteriaceae and LAB showed a high correlation to the storage time, especially at higher temperatures. However, this correlation is less pronounced for lower temperatures. The physicochemical parameters pH, color and texture showed lower significant correlations to the storage time (r < 0.8), depending on the respective storage temperature (Table 2). Since no reliable and reproducible correlation to storage time was observed for the investigated storage temperatures, these parameters were rejected for the choice as spoilage predictors. The oxygen content showed a high correlation to the storage temperature in all trials, but changes in atmosphere occurred mostly after the end of shelf-life. Since highest changes in the atmosphere within the packaging were observed after the samples were judged inacceptable from a microbial or sensory point of view (data not shown), this parameter was also discarded as spoilage predictor. As sensory investigations as well as TPC showed the highest correlation to storage time and most robust response during all investigated temperatures, these parameters were chosen for further modeling.

**Table 3:** Correlation of the different microbial parameters and the storage time of ma-packed pork loins under different isothermal conditions (Spearman’s rho).

	Microbial Parameters						
Temperature [°C]	TPC	TVC	PSE	*B. thermosphacta*	Enterobacteriaceae	LAB	Yeasts/Molds
2	0.923 **	0.828 **	0.541 **			0.866 **	0.659 **
4	0.926 **	0.816 **	0.727 **			0.771 **	0.789 **
7	0.894 **	0.892 **	0.916 **	0.883 **	0.674 **	0.891 **	0.925 **
10	0.913 **	0.900 **	0.666 **		0.485 **	0.868 **	0.766 **
15	0.930 **	0.912 **	0.931 **	0.895 **	0.869 **	0.910 **	0.922 **
25	0.929 **	0.944 **	0.872 **	0.831 **	0.905 **	0.938 **	0.922 **

The correlation coefficient r is indicated only for significant correlations with ** meaning significant at the 0.01-level.

### 3.2. Shelf-Life Modeling

**Figure 3:**
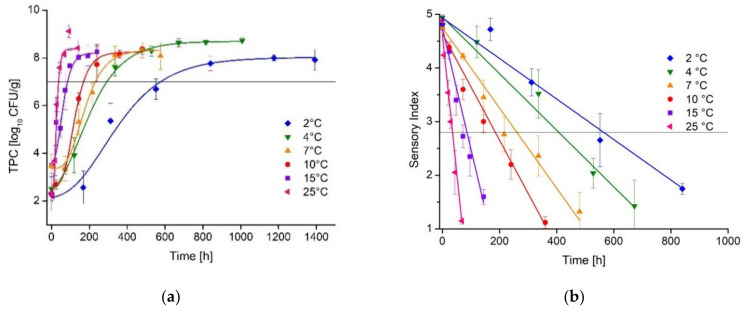
(**a**) Growth kinetics of the total psychrotroph count on ma-packed pork loin stored at different isothermal conditions (mean ± standard deviation, *n* = 362). All growth curves fitted with modified Gompertz equation. (**b**) Development of the sensory index on ma-packed pork loin stored under different isothermal conditions (mean ± standard deviation, *n* = 227), data fitted by linear regression.

For the primary level model, the development of TPC and SI were fitted as a function of time for all isothermal storage tests (Trial A) at 2, 4, 7, 10, 15 and 25 °C (Figure 3). For primary modeling of TPC, the 4 °C references of the dynamic trials (B0, C0, D0) were included in the data set to improve the prediction. Microbial as well as sensory models showed a good model performance with a R^2^ between 0.96 and 0.99. The estimated microbial shelf-life ranged between 576 h (2 °C) and 37 h (25 °C). The microbial shelf-life for the recommended storage temperature of 7 °C was 232 h. Microbial growth parameters reflecting the spoilage kinetics are displayed in Appendix A Table A1.

**Figure A1:**
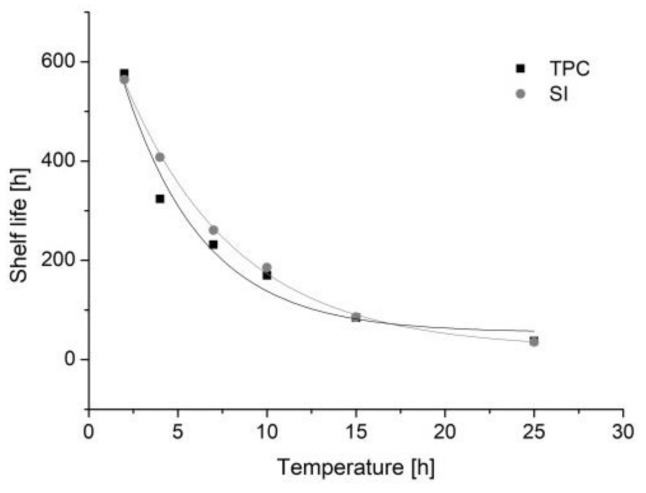
Contour plot of the shelf-life estimated via microbial and sensory parameters.

The estimated sensory shelf-life ranged between 564 h (2 °C) and 35 h (25 °C) with a shelf-life of 261 h at the recommended storage temperature of 7 °C. An overview on sensory decay parameters is given in Table A2. A contour plot for the dependency of microbial and sensory shelf-life on temperature is given in Figure A1.

**Figure A2:**
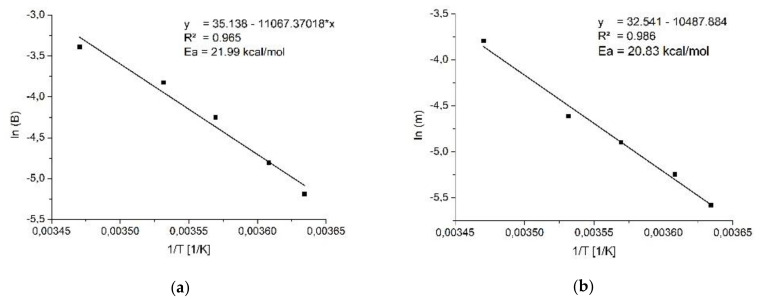
Secondary modeling for (**a**) microbial and (**b**) sensory spoilage kinetics.

For the secondary level model, the relative growth rate of TPC as well as the slope of the SI (kinetics of the sensory decay) was modeled as a function of temperature using the Arrhenius equation (Figure A2). For this approach, results of the storage temperature of 25 °C were excluded to avoid inaccurate predictions in the lower temperature range. However, the range between 2 °C and 15 °C is crucial for transportation and storage of fresh meat in practice. The model performance showed a good data fit with a R^2^ of 0.965 for microbial and R^2^ of 0.986 for sensory data.

### 3.3. Validation of the Shelf-Life Models under Laboratory and Practical Conditions

**Figure 4:**
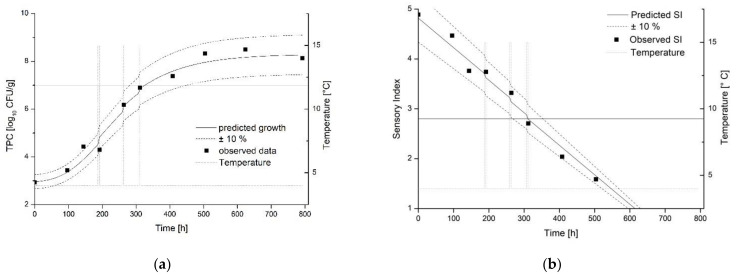
Validation of the model under dynamic temperature conditions (C1) by comparing the observed (points) and predicted (lines) spoilage of ma-packed pork loin. (**a**) Growth of TPC. (**b**) Sensory data. Storage scenario (C1).

The validation of the dynamic shelf-life model under laboratory conditions revealed that predictions are most suitable for scenarios with temperature shifts in the exponential growth phase (Trial C1, see Table 1 for temperature profiles). Figure 4 displays the dynamic modeling of TPC growth (Figure 4a) and development of the SI (Figure 4b) in dependency on non-isothermal temperature conditions.

This is reflected by the validation coefficients displayed in Table 4. The microbial as well as sensory shelf-life model showed lowest RMSE at Scenario C1 and most accurate predictions according to the Bf, Af and R^2^. Similarly, the actually calculated shelf-life and the prediction showed the least deviation in this scenario. The microbiological model resulted in a slight underestimation of 0.4% to the calculated shelf-life. In both cases, the shelf-life is calculated to 13 days. For the sensory model, there is a slight underestimation of 2.7% with a shelf-life of 310 h, which corresponds to a shelf-life of 12 days.

Temperature shifts during the lag phase of microbial growth led to an underestimation of microbial growth (Trial B1, data not shown). This is reflected by the highest RMSE and lowest R^2^ for the microbial as well as sensory prediction. The Bf under one points to a slight under prediction of microbial as well as sensory development. Validation indices for Trial D1 are comparable to Trial B1, but the prediction of the SI was less accurate according to the Bf and Af. For both trials, the prediction shows an aberration of 0.30–0.47 log_10_ CFU/g according to the RMSE. In addition, higher deviations occur both in the calculated and between the calculated and predicted shelf-lives of the sensory and microbiological data. The predictions of both models differ by 4.8% for Model B1 and by 4.5% for Model D1.

**Table 4:** Validation indices for the dynamic modeling of microbial and sensory development.

Scenario	RMSE	Bf	Af	R^2^
Microbial development				
B1	0.47	0.99	1.09	0.95
C1	0.30	0.99	1.04	0.98
D1	0.40	1.00	1.07	0.96
Sensory development				
B1	0.51	0.91	1.18	0.83
C1	0.15	1.01	1.05	0.98
D1	0.51	0.80	1.25	0.87

There are different factors influencing the performance of the dynamic model. First, different microbial contamination on the samples and variability of microbial growth can lead to uncertainties of shelf-life prediction [5]. The initial contamination can vary significantly between different batches (data not shown). Additionally, the dynamic model is optimized for microbial growth during the exponential phase since it relies on the temperature-dependent prediction of the microbial growth rate. As a result, the growth during the lag phase is underrepresented in the model and temperature shifts in the lag phase are not modeled as accurately as temperature shifts during the exponential phase. Temperature shifts in the lag phase have a stronger effect on microbial growth in comparison to shifts in the exponential phase, as it was already shown in former studies [14]. Further developments are needed for optimizing dynamic models to minimize these uncertainties.

### 3.4. Development of the DSLC

**Figure 5:**
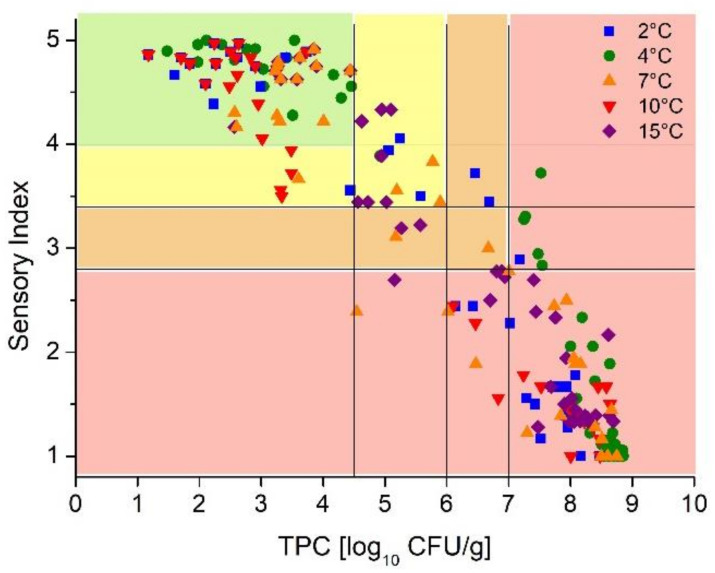
Correlation between the results of the microbial and sensory evaluation. Classification of the DSLC.

The developed models for the prediction of microbial and sensory data were combined to establish an indicator as dynamic shelf-life criterion. The SI was plotted against the TPC and quality levels were defined based on a four-point scoring system (Figure 5). The graph shows the high variability of microbial and sensory data in the complete temperature range. Thus, the scoring system of the DSLC was developed with respect to an appropriate match of microbial and sensory data.

The highest score represents the highest sensory and microbial quality of the product. The microbial acceptance level was defined as 7 log_10_ CFU/g according to the ICFMS and the sensory acceptance level was defined as 2.8 according to the applied scheme. An overview of the levels used is presented in Table 5.

The scoring system is an easy-to-use indicator combining an accurate real-time prediction with a simple way to communicate the product status to all actors in the chain. By knowing the spoilage kinetics of the specific product, the DSLC can easily be adapted to different fresh food products.

### 3.5. Pilot Study and Implementation of the DSLC

The implementation of dynamic modeling and the DSLC under real chain conditions was challenging and characterized by more inaccuracies in prediction, in comparison to the laboratory validation studies. The highest RMSE was observed for the prediction of the microbial development in Trial E1 (Table 6). For this scenario, an under prediction of microbial growth and aberration of 9% was observed according to the Bf and Af. The predicted microbial shelf-life was 376 h (15.6 days) while the predicted sensory shelf-life was 396 h (16.5 days). Even though both predicted shelf-lives were in good accordance, the difference to the observed shelf-life (Δ 3 days) was notable.

**Figure 6:**
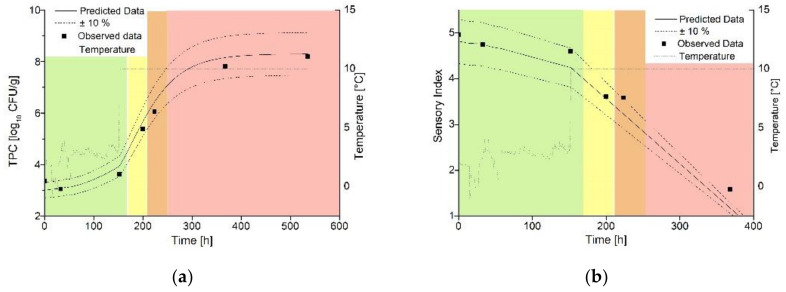
Validation of the model in a pilot study (E2) by comparing the observed (points) and predicted (lines) spoilage of ma-packed pork loin. (**a**) Growth of TPC. (**b**) Sensory date.

The real-time prediction of the microbial and sensory development was more accurate for scenario E2, with a RMSE of 0.28 and 0.27, respectively. Additionally, Af, Bf and R^2^ represented a better model performance for Scenario E2. The predicted shelf-life was 246 h (10.3 days, microbial) and 254 h (10.6 days, sensory), respectively. The observed shelf-life was 264 h (11.0 days, microbial) and 267 h (11.1 days, sensory), respectively. The microbial and sensory data as well as the dynamic modeling approach for Trial E2 is displayed in Figure 6. Additionally, the scoring of the DSLC is highlighted. According to the prediction, the product can be judged with Score 4 until 169 h according to microbial and sensory prediction as well. DSLC Score 3 is valid until 210 h (microbial prediction) and 211 h (sensory prediction). Finally, the end limit of Score 2 for the product, which is equivalent to the end of shelf-life, is achieved after 247 h (microbial prediction) and 254 h (sensory prediction). For the practical implementation, a mean value of microbial and sensory prediction can be calculated to generate the time when the limits of the DSLC will be achieved.

For the implementation in real supply chains, a specific adjustment to the product kinetics and the needs of the actors in the chain is crucial [17]. Based on predictive modeling and the combination with temperature monitoring, dynamic shelf-life decision systems (SLDS) can be integrated to optimize quality management in the supply chain [34,35]. By incorporation into the supply chain, the DSLC offers the opportunity to efficiently make use of the sales window and optimize logistic processes or adjust the storage temperatures [19,36]. Additionally, real-time modeling of the DSLC offers the opportunity to implement dynamic pricing in order to reduce food waste as it was proposed by former studies [3].

Even though the DSLC showed a very accurate prediction of the product status within the supply chain of Trial E2, the inaccuracies in the prediction during the pilot study E1 are not satisfying. Since both pilot studies were conducted within the same batch and initial TPC of the samples was similar, different levels of microbial contamination is not considered a reason for the aberrations in prediction during Trial E1. Microorganism during this trial showed a growth velocity comparable to the 10 °C isothermal trials rather than the 4 °C effective temperature observed in the supply chain. Microbial contamination on meat varies largely in terms of the number of microorganisms as well as the species composition. This can lead to microbial interaction processes and differing enzyme activity during spoilage [7]. Within this study, the microflora of the samples comprised mainly LAB, PSE and *B. thermosphacta* at lower temperatures. Some LAB are known for their bacteriocinogenic potential [37], and thus, inhibition processes are one further possible explanation for variation in the spoilage process. Since the model is built on TPC, it is not sensitive for those processes related to specific species or species groups. However, the more general prediction offers the advantage of suitability for different products and independence from microbial diversity originating in different primary production characteristics. A solution for this problem would be the measurement of the microbial contamination in real-time, as is possible by rapid detection methods [33]. The implementation of rapid detection methods in existing quality managements systems could thus improve the accuracy of prediction by integrating information about the microbial contamination of the product. Additionally, the biological variation of microbial growth could be monitored in real-time to correct predictive models and improve the model performance. These are promising steps to overcome some of the last challenges of implementation.

## 4. Conclusions

The DSLC, in combination with adequate temperature monitoring solutions, is considered an effective tool to reflect the actual status of the product. The combination of microbial and sensory parameters for dynamic modeling as well as the replication on the DSLC is an easy but sophisticated way for a simplified description of the complex spoilage processes occurring in fresh ma-packed pork meat. It can easily be adapted to other perishable foods and can contribute to an improved cold chain management and long-term prevention of food waste. Even though the concept for the real-time prediction of the status and remaining shelf-life of the product was validated successfully in pilot studies, some inaccuracies in the models are still left. Variation of the microbial contamination of the product and under prediction of microbial growth at temperature violations during the lag phase remain problems affecting the accuracy of prediction. Thus, implementing rapid detection methods as a tool to correct predictive models is one main challenge and opportunity for the future.

## Figures and Tables

**Table 1:** Overview of the investigated constant and dynamic storage scenarios.

Trial	Scenario	Description of Storage Conditions
Trial A (*n* = 262)	A1	A constant storage temperature at 2 °C
	A2	A constant storage temperature at 4 °C
	A3	A constant storage temperature at 7 °C
	A4	A constant storage temperature at 10 °C
	A5	A constant storage temperature at 15 °C
	A6	A constant storage temperature at 25 °C
Trial B (*n* = 90)	B0	A constant storage temperature at 4 °C
	B1	4 shifts for 4 h from 4 to 15 °C at the beginning of storage
Trial C (*n* = 95)	C0	A constant storage temperature at 4 °C
	C1	4 shifts for 4 h from 4 to 15 °C during the exponential phase
Trial D (*n* = 120)	D1	A constant storage temperature at 4 °C
	D2	3 shifts to 15 °C after 4, 24 and 48 h and an increase to 7 °C after 120 h till the end of the storage
Trial E (*n* = 80)	E1	Validation study under practical conditions with a basic storage temperature of 4 °C
	E2	Validation study under practical conditions with a basic storage temperature of 10 °C

**Table 5:** Sensory and microbial acceptance levels for the development of the DSLC.

DSLC Acceptance Level	4	3	2	1
TPC log_10_ CFU/g	<4.5	4.5–6	6–7	>7
SI	>4	4–3.4	3.4–2.8	<2.8

**Table 6:** Validation indices of microbial and sensory models during the pilot studies.

Scenario	RMSE	Bf	Af	R^2^
Microbial development				
E1	0.60	0.94	1.09	0.92
E2	0.28	1.02	1.05	0.98
Sensory development				
E1	0.24	0.97	1.07	0.97
E2	0.27	0.92	1.09	0.95

## Data Availability

The data are available upon request.

## Appendix A

**Table A1:** Growth parameters of the TPC on ma-packed pork loin under different isothermal conditions.

Temperature [°C]	A [log_10_ CFU/g]	B [1/h]	M [h]	C [log_10_ CFU/g]	R²
2	2.1 (±0.6)	0.0056 (±0.001)	278 (±50)	5.9 (±0.6)	0.977
4	3.0 (±0.4)	0.0075 (±0.001)	186 (±24)	5.7 (±0.5)	0.985
7	3.4 (±0.3)	0.014 (±0.003)	150 (±13)	5.0 (±0.3)	0.985
10	2.6 (±0.1)	0.022 (±0.002)	106 (±3)	5.6 (±0.1)	0.998
15	3.6 (±0.4)	0.034 (±0.010)	49 (±6)	4.6 (±0.5)	0.975
25	2.2 (±0.4)	0.086 (±0.030)	21 (±4)	6.2 (±0.5)	0.976

**Table A2:** Parameters of the linear regression of the sensory index on ma-packed pork loin under different isothermal conditions.

Temperature [°C]	Slope	Intercept	R^2^
2	−0.00378 (±0.00023)	4.93 (±0.14)	0.985
4	−0.00527 (±0.00026)	4.95 (±0.03)	0.990
7	−0.00747 (±0.00046)	4.75 (±0.05)	0.982
10	−0.00992 (±0.00048)	4.64 (±0.10)	0.988
15	−0.02254 (±0.00138)	4.74 (±0.10)	0.982
25	−0.05122 (±0.00220)	4.61 (±0.10)	0.991
